# Spatial and temporal analysis of surface hardness across a third-generation artificial turf pitch over a year

**DOI:** 10.1177/1754337114523756

**Published:** 2014-09

**Authors:** Stephanie E Forrester, Felix Tsui

**Affiliations:** Wolfson School of Mechanical and Manufacturing Engineering, Loughborough University, Loughborough, UK

**Keywords:** Artificial turf, hardness, Clegg hammer, spatial variability, temporal variability

## Abstract

Despite the potentially negative effects on play performance and safety, little is currently known about the spatial and temporal variability in the properties of artificial turf pitches. The primary purpose of this study was to quantify the spatial and temporal variations in surface hardness across a 5-year-old third-generation artificial turf pitch over full year cycle. The secondary purpose was to investigate the key variables that contributed to these variations in surface hardness using a correlation approach. Surface hardness (2.25 kg Clegg impact hammer, average of drops 2–5), ground temperature and infill depth were measured at 91 locations across the third-generation artificial turf pitch in 13-monthly test sessions from August 2011 to August 2012 inclusive. For each month, rainfall in the 24 h prior to testing and pitch usage statistics were also obtained. Shockpad thickness was obtained from measurements taken when the carpet was replaced in 2007. Spatial and temporal variations were assessed using robust statistical measures while Spearman correlation was used to assess the contributions of the secondary variables to surface hardness variability. The results indicated that spatial variation in surface hardness exceeded temporal variation; the former demonstrated a median absolute deviation of 12 ± 1 G across the pitch in any test session while the median absolute deviation for the latter was only 4 ± 2 G across the 13 test sessions. Spatial variation in surface hardness was moderately correlated with shockpad thickness and weakly correlated with infill depth (both negative). These results reinforce the importance of monitoring spatial and temporal variations in play performance variables for third-generation surfaces as well as providing support for the role of maintenance in minimising the spatial variation.

## Introduction

**Figure 1.**
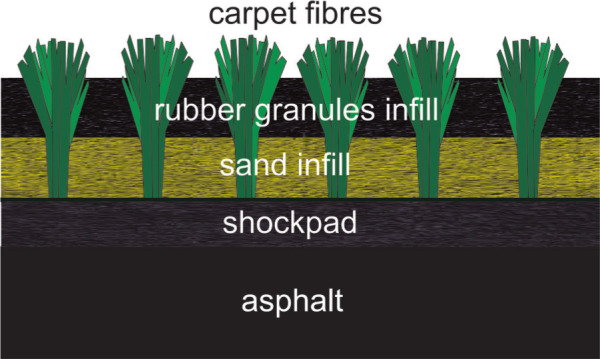
Schematic of a 3G artificial turf system. The upper carpet layer comprises 40- to 65-mm-long monofilament or fibrillated fibres with a sand (stabilising) and rubber crumb (shock absorption) infill. The lower shockpad layer is commonly included to increase the shock absorption properties of the system.

Artificial turf (AT) surfaces, in particular third generation (3G), are becoming increasingly popular in sports facilities across the United Kingdom^1^ as well as more globally due to climatic changes and urban growth in many countries. In brief, the 3G carpet layer has a longer pile length, less abrasive fibres, greater infill depth and lower tuft density compared to earlier generations^1^ (Figure 1). The 3G system was originally designed for football and aims to emulate the play performance, that is, ball–surface and player–surface interactions, of the game played on natural grass, yet provide a more durable and consistent surface than the latter. The newest 3G pitches can also facilitate other sports such as hockey, lacrosse, rugby union, rugby league, Gaelic football and Australian Rules football. The fundamental play performance of AT pitches is dependent on the design and installation of the surface system and principally by the properties and interactions between the components comprising the shockpad and carpet layers (Figure 1). In situ play performance also depends on levels of usage and maintenance as well as environmental and climatic factors such as ground temperature, moisture content and contamination (e.g. both organic matter such as foliage from surrounding trees and inorganic matter such as fractured fibres) within the system.^2^ It is typically characterised through a series of mechanical tests, for example, Fédération Internationale de Football Association (FIFA),^3^ which provide standards for player–surface and ball–surface interactions as well as pitch durability, which aim to ensure that the surface is consistent and appropriate for the demands of the sport(s).

Although AT pitches tend to be very uniform in appearance, spatial and temporal variations in play performance can exist through various mechanisms principally related to the shockpad and carpet layers.^4^ Spatial variation can exist due to the following: surface system design or installation leading to inconsistencies in shockpad thickness, infill depth and/or sand–rubber ratio; player loading leading to infill compaction, loss and/or migration from high- to low-use areas;^4–7^ lack of appropriate maintenance and localised contamination, for example, foliage from surrounding trees. Spatial variation in the behaviour of sports surfaces is undesirable as it can lead to issues with play performance; it is suggested to be a causal mechanism in injury occurrence^8^ and results in unpredictable ball behaviour threatening the quality and enjoyment of the game. Further evidence for the importance of controlling spatial variation is the recent increase in the number of field test locations for shock absorption and vertical deformation, from 6 to 19, within the FIFA Quality Concept for Football Turf.^3^ In contrast, temporal variation in play performance can exist due to climatic factors, such as temperature effects on the properties of the system components, and rainfall affecting the interaction behaviour between the carpet fibres and infill materials. At present, there is a lack of information and understanding relating to typical magnitudes and contributions of spatial and temporal variations to play performance of 3G AT systems.

Surface hardness represents one of the key play performance variables and for player–surface interactions is typically quantified using the Advanced Artificial Athlete^3^ (AAA) or the Clegg impact hammer^9^ (CIH). For a 3G AT, pitch surface hardness is largely determined by the current state of the rubber infill (and to a lesser extent the sand infill) and shockpad layer and can vary both spatially and temporally due to the reasons outlined above. Surface hardness has been shown to influence a player’s perception of an AT pitch^10^ and to affect the mechanics of the player–surface interaction. Potthast^11^ found surface hardness to affect technique during an approach and kicking movement; it was also suggested that ball rebound and overall gameplay can also be affected. Numerous biomechanical studies have shown that surface stiffness affects the mechanics of running where humans adjust their leg stiffness based on the surface stiffness in order to stabilise their vertical centre-of-mass movement.^12,13^ Surface hardness is also thought to be relevant to player safety^14,15^ with the International Rugby Board (IRB) regulations for AT in rugby union including a head impact criteria test to ensure player safety.^16^ Thus, understanding the spatial and temporal variations in surface hardness and the factors that influence these are important components in optimising AT surfaces from play performance and safety perspectives.

The primary purpose of this study was to quantify the spatial and temporal variations in surface hardness across a 5-year-old 3G AT pitch over a full year cycle (13 months). A secondary purpose was to investigate the key variables that contributed to the observed variations using a correlation approach. For spatial variation in surface hardness, the variables included shockpad thickness, infill depth and ground temperature; whilst for temporal variation, the variables included infill depth, ground temperature, rainfall, usage and air temperature. It is anticipated that the results gained can be used to provide recommendations for minimising the spatial and temporal variations in surface hardness for 3G AT pitches.

## Methods

### Pitch history

The 3G AT pitch selected for this study was a multi-purpose facility at Loughborough University used for a range of sports principally being football, rugby union, hockey and lacrosse. The pitch covers an overall area of 107 m × 62.5 m. It was originally a sand-based facility with a macadam base and a 25-mm rubber shockpad laid in situ. In 2007, the carpet was replaced with a 3G system and the shockpad repaired. Shockpad thickness at 60 locations across the pitch was measured and recorded at this time and these data were used in this study. The thickness was 24.4 ± 4.9 mm (mean ± standard deviation (SD)) with a range of 13–38 mm following the repairs with 43 of the 60 locations falling within 1 SD of the mean. The 3G carpet consists of 35-mm monofilament polyethylene fibres with an infill of silica sand, depth 10–12 mm, and styrene butadiene rubber (SBR), depth 12–15 mm. Throughout the study the pitch was brushed once a week to de-compact the infill and cleaned monthly for contaminants using a Hörger rotary brush. The pitch is heavily used (up to 50 h per week) during University term, primarily by students for training, and more intermittently used at other times.

### Pitch testing

The properties of the pitch were subjected to 13 test sessions, approximately 1 month apart, from August 2011 to August 2012 inclusive. Each session was no less than 3 weeks and no more than 5 weeks apart and was arranged based on weekly maintenance schedules provided by the facilities management team at Loughborough University. This ensured that testing always occurred after the monthly maintenance brushing. In each session, the same two trained operators were responsible for all measurements. Surface hardness was measured at 91 locations across the pitch using a 2.25-kg CIH dropped from a height of 0.45 m. This device provided the peak deceleration (expressed in gravities, G) on impact with the surface, where higher peak decelerations indicated a harder surface. Each test location was subjected to five consecutive impacts and an average of the last four impacts was used to provide hardness for the location. Infill depth (in millimetres) was measured three times around each surface hardness location using a mechanical depth gauge meter and ground temperature (°C) was measured at each location using a temperature probe inserted into the rubber infill layer. Total rainfall (in millimetres) in the 24 h prior to surface testing and air temperature during testing were obtained from the local weather station (Mountsorrel), while usage statistics for the pitch were also obtained from the booking records of the Sports Development Centre at the University in terms of hours booked per calendar month. Usage for any given test session was then determined as the hours of use per month since the last test session

(1) $$\begin{array}{c} \mathrm{usage}\;(\mathrm{hours}\;\cdot \;\mathrm{mont}h^{-1})\\ =\frac{\left\{\sum_{\begin{array}{c} \mathrm{contributing}\\ \mathrm{months} \end{array}}\left(\frac{n_{\mathrm{days}}}{n_{\mathrm{total}}}\right)\times t_{\mathrm{bookings}}\right\}}{\sum_{\begin{array}{c} \mathrm{contributing}\\ \mathrm{months} \end{array}}\left(\frac{n_{\mathrm{days}}}{n_{\mathrm{total}}}\right)} \end{array}$$

where *n_days_* is the number of days from a calendar month that contributed to the evaluation period, *n_total_* is the total days in the calendar month and *t_bookings_* is the number of hours booked in the calendar month.

### Statistical analysis

Normality of all variables was first tested, and rejected, using the Shapiro–Wilk test (*p*≤ 0.05). Consequently, robust statistics and non-parametric tests were selected for further analysis of the data. Analysis of the pitch data was then performed in three stages: descriptive statistics to quantify central tendency and variability; significance testing to analyse for changes over time and correlation analysis to test for relationships between measured variables and surface hardness. All data analysis was performed using MATLAB R2010a (The MathWorks, Inc., Natick, MA, USA) with a significance level of *p*≤ 0.05.

In stage 1, descriptive statistics for the spatial variation in surface hardness, ground temperature and infill depth were quantified by the median and variability about this value represented by the median absolute deviation (MAD), inter-quartile range (IQR) and robust coefficient of variation (rCV = 100% × 0.7413 × IQR/median) evaluated using all test locations for each test session. These statistics were then summarised using the median and IQR deviation for the 13 test sessions considered. Temporal variation was quantified by the same statistical parameters evaluated using all test sessions for each test location. These statistics were again summarised using the median and IQR for the 91 test locations considered.

In stage 2, differences in surface hardness, ground temperature and infill depth over the 13-monthly test sessions were analysed using the one-way Kruskal–Wallis test with Dunn’s multiple comparisons test.

**Figure 2.**
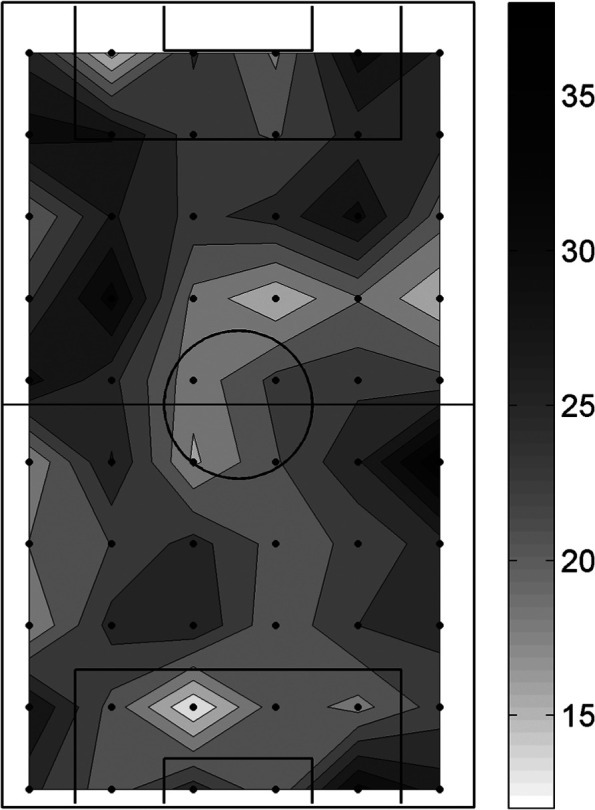
Contour map of shockpad thickness (mm) across the pitch determined from data obtained at the time of pitch repair in 2007. The black dots represent the 60 locations where shockpad thickness measurements were taken.

In stage 3, the relationship between spatial variation in surface hardness and shockpad thickness, infill depth and ground temperature, was examined using Spearman’s correlation on the test session averaged data from the individual test locations across the pitch. Shockpad thickness data were available at 60 locations over the pitch and were interpolated to the same locations as used for the surface hardness, infill depth and ground temperature measurements (Figure 2). The relationship between temporal variation in surface hardness and temporal variation in each of ground temperature, infill depth, rainfall, usage and air temperature was also examined using Spearman’s correlation on the test location averaged data from the individual test sessions.

## Results

### Descriptive statistics

**Figure 3.**
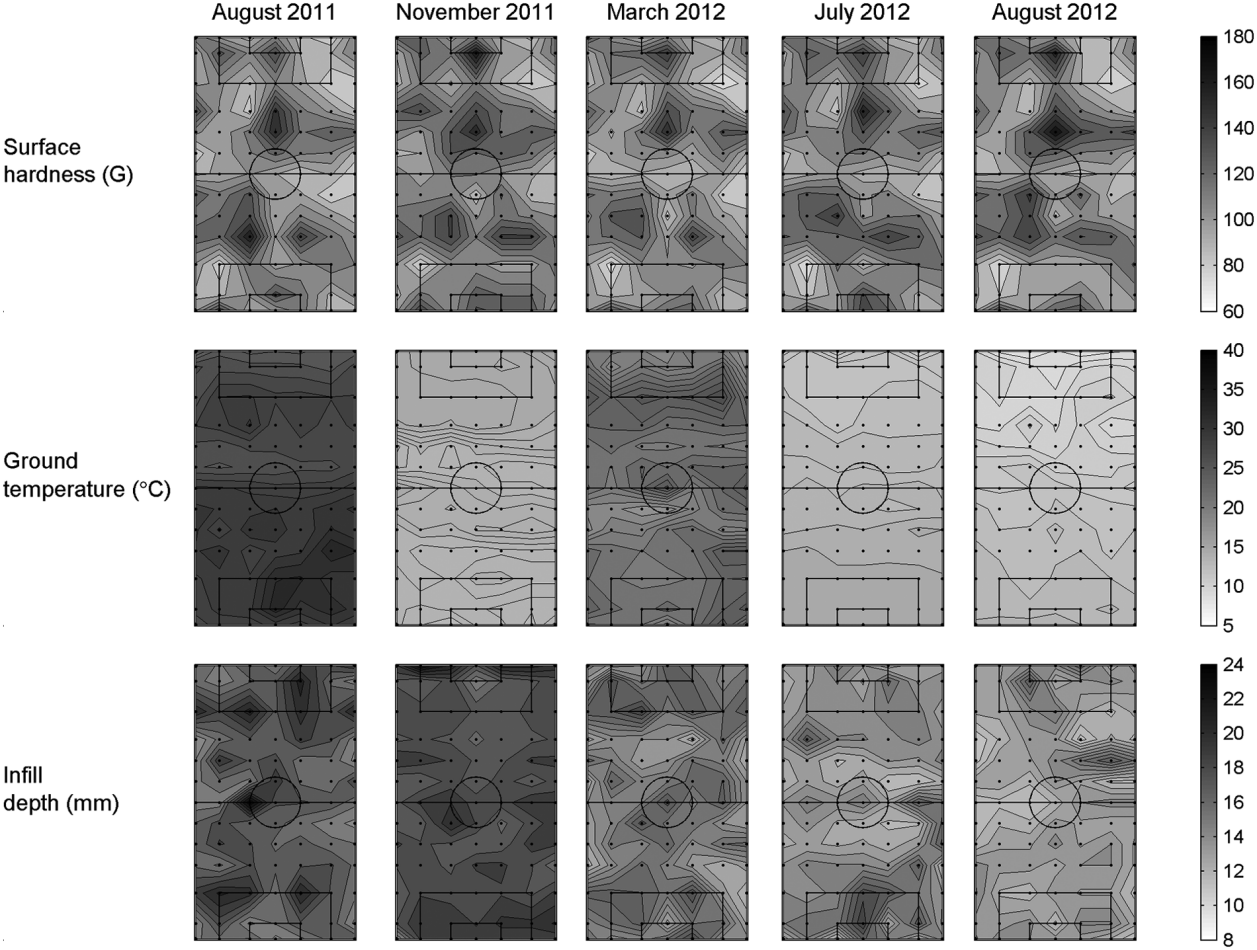
Contour maps of surface hardness (G), ground temperature (°C) and infill depth (mm) from five of the test sessions. The black dots represent the 91 locations where measurements were taken.

The spatial and temporal variations in surface hardness, ground temperature and infill depth are summarised in Table 1. For surface hardness, the spatial variation corresponded to a range of ∼80 G (from 77 to 157 G) across the pitch in any test session, while the temporal variation was less than half this with a range of ∼20 G (from 94 to 115 G) for the pitch across test sessions. For ground temperature, the spatial variation corresponded to a range of ∼8 °C (from 15.6 °C to 23.7 °C) across the pitch in any test session, while the temporal variation was over twice this with a range of ∼20 °C (from 11.6 °C to 31.2 °C) for the pitch across test sessions. For infill depth, the spatial variation corresponded to a range of ∼8 mm (from 12.3 to 19.8 mm) across the pitch in any test session, while the temporal variation was only slightly lower with a range of ∼5 mm (from 13.4 to 18.7 mm) for the pitch across test sessions. The contour maps in Figure 3 provide visual support for these observations; notably the larger spatial variation in surface hardness compared to the temporal variation, but small spatial variation in ground temperature compared to the temporal variation.

**Table 1.** Median and IQR of the descriptive statistics for surface hardness (G), ground temperature (°C) and infill depth (mm) determined (1) spatially evaluated over the 91 test locations for each test session and (2) temporally over the 13-monthly test sessions for each test location. Note that the data for month 5 were neglected as the pitch was frozen and only surface hardness could be measured.

		Surface hardness				Ground temperature				Infill depth			
		Median (G)	MAD (G)	IQR (G)	rCV (%)	Median (°C)	MAD (°C)	IQR (°C)	rCV (%)	Median (mm)	MAD (mm)	IQR (mm)	rCV (%)
Spatial	Median	108	12	24	16.3	19.6	1.1	2.1	7.7	15.0	1.0	1.8	8.2
	IQR	5	1	3	2.1	10.9	0.5	0.7	3.8	3.1	0.3	0.6	3.6
Temporal	Median	107	4	8	5.3	19.6	5.9	11.4	42.0	15.4	1.7	3.4	16.6
	IQR	23	2	4	2.8	1.6	1.0	1.3	3.8	0.8	0.5	0.8	3.7

MAD: median absolute deviation; IQR: inter-quartile range; rCV: robust coefficient of variation.

### Significance testing

**Figure 4.**
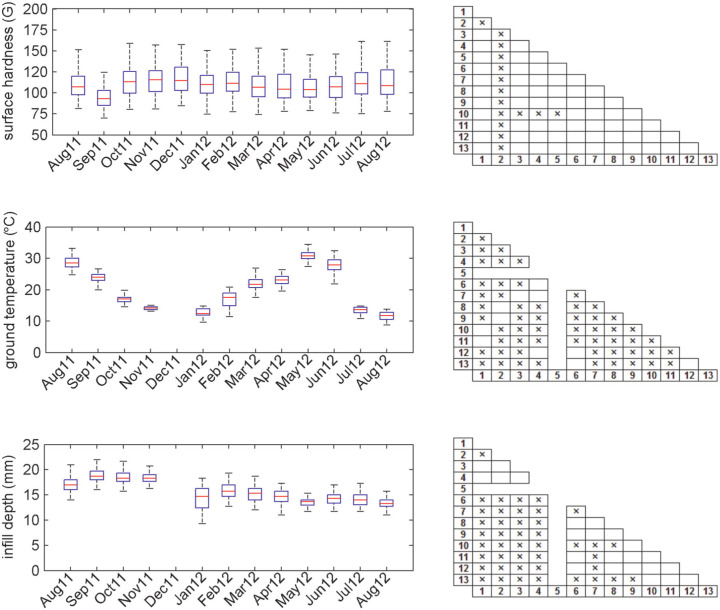
Boxplots for surface hardness, ground temperature and infill depth for each of the 13-monthly test sessions. The central red line is the median; the edges of the box represent the IQR and the black errors extend to the extremes of the data (excluding outliers). Also shown in the right-hand grids are where significant differences existed between specific test sessions, represented by the ‘×’ boxes. Note that data for month 5 were incomplete as the pitch was frozen and only surface hardness could be measured.

The significant differences in surface hardness primarily occurred in month 2 (September 2011) where the hardness was significantly lower than in all other months (Figure 4). In comparison, for ground temperature the majority of months were significantly different to the majority of other months, reflecting the seasonal changes in ground temperature. For infill depth, the principal differences were between months 1 and 4 (August 2011 to November 2011) where the infill depths were greater than the remaining months 6–13 (January 2012 to August 2012).

### Correlation analysis

Spatial variation in surface hardness showed a moderate negative correlation with shockpad thickness (r = −0.477, *p* < 0.001) and a weak negative correlation with infill depth (r = −0.229 *p* = 0.029); however, there was no correlation with ground temperature (Table 2). Temporal variation in surface hardness showed no significant correlation with any of the temporal variables (Table 2).

**Table 2.** Spearman correlation coefficient results for (1) spatial variation in surface hardness with ground temperature, infill depth and shockpad thickness and (2) temporal variation in surface hardness with ground temperature, infill depth, usage, rainfall and air temperature.

		Spearman correlation coefficient, r_S_ (−)	*p* (−)	Effect size
Spatial	Ground temperature	−0.071	0.505	–
	Infill depth	−0.229	0.029	Small
	Shockpad thickness	−0.477	<0.001	Medium
Temporal	Ground temperature	−0.567	0.055	Large
	Infill depth	−0.044	0.892	–
	Usage	0.387	0.214	–
	Rainfall	−0.319	0.312	–
	Air temperature	−0.091	0.779	–

Notes: effect size was assumed to be large for ∣r_S_∣≥ 0.50, medium for 0.50 > ∣r_S_∣≥ 0.30 and small for ∣r_S_∣ < 0.30.^22^

## Discussion

The primary purpose of this study was to quantify the spatial and temporal variations in surface hardness across an existing 5-year-old 3G AT pitch over a full year cycle. The results showed that spatial variability in surface hardness exceeded the temporal variability; the former demonstrated approximately a 2-fold range in surface hardness across the pitch at any point in time (range 77–157 G), while the latter was less than half this (range 94–115 G). A secondary purpose was to investigate the key variables that may have contributed to these variations in terms of how well they correlated with surface hardness. Spatial variation in surface hardness was moderately correlated with shockpad thickness (r = −0.477) and weakly correlated with infill depth (r = −0.229). These results are unsurprising given that the shockpad layer and (rubber) infill are the components designed to provide the shock-absorbing properties of the surface system. Somewhat surprisingly, surface hardness failed to show a correlation with the remaining temporal variables considered (infill depth, usage, rainfall and air temperature). For infill depth, this is most likely due to the small range in values recorded, from 18.7 mm in month 2 (September 2011) down to 13.4 mm in month 13 (August 2012).

As noted in the section ‘Introduction’, the shockpad layer and rubber infill are the main system components intended to provide the shock-absorbing properties of the pitch. Although the 3G pitch was only 5 years old, the shockpad was substantially older having been laid in 1998 when the original sand-based AT facility was installed. The shockpad was repaired when the 3G system was installed in 2007 and these repairs, combined with its age, will have contributed to the inconsistent thickness (mean ± SD of 24.4 ± 4.9 mm and range of 13–38 mm; Figure 2) and, hence, the spatial variation in surface hardness. Although this spatial variation in shockpad thickness appears large, it is uncertain whether it can be classified as atypical due to a lack of data reported on this topic. Most in situ shockpads will demonstrate some level of spatial variation in thickness as this layer is commonly used to even out any inconsistencies in the lower layers and this can be further exasperated through use and repair. The absolute infill depths were, on average, ∼10 mm lower than the infill depth at installation (see section ‘Methods’). Part of this difference may have been due to inter-operator differences in measurement technique due to the subjectivity in judging when the depth gauge had fully penetrated the infill layer to the carpet backing. However, it also suggests that infill loss, redistribution and/or compaction had occurred, all of which can contribute to spatial variation in surface hardness. This result reinforces the role of maintenance, particularly the processes that target decompacting, redistributing and topping up infill across the pitch, in order to maximise the consistency of play performance characteristics.

In general, ground temperature showed the greatest amount of temporal (seasonal) variation and only small spatial variation. The low spatial variation is unsurprising and that observed will, in part, be influenced by the measurement techniques where it took on average 3 h to complete the measurements across the pitch. The magnitude of the IQR boxes in Figure 4 suggests that ground temperature did vary substantially in some test sessions, notably February 2011 and June 2012; however, this variation was much lower than the temporal variation and did not affect the surface hardness measurements. The strong negative correlation between temporal ground temperature and surface hardness data suggests that as ground temperature dropped, the pitch became harder, with the extreme being freezing temperatures, which in this study occurred in month 5 (December 2011). This resulted in the second highest measured median pitch hardness of 115 G, with the pitch frozen to such an extent that ground temperature and infill depth measurements could not be taken.

The weather station data and usage statistics did not correlate with the temporal variation in surface hardness. The weather station results were perhaps unsurprising given that rainfall was being used as an indirect estimate of the pitch moisture content; at present, no direct validated method exists to measure this quantity. The accuracy of this estimation is unknown as are the effects of moisture on surface hardness. The usage statistics results are perhaps more surprising as it may have been expected that when the pitch had been more heavily used, it would have been harder, primarily through infill compaction and migration. However, this pitch is used for both organised team practice and ad hoc play at other times; the usage data captured only considered the former. Furthermore, the usage data do not account for how many players were involved in each booked session, the sport being played or how the pitch was being used, for example, as a full-sized pitch or multiple smaller cross-field pitches. All these factors are likely to affect the resulting mechanical wear experienced by the pitch but are difficult to record on a long-term basis and highlight the challenges in quantifying pitch usage. The usage statistics used in this study may simply not have been sufficiently precise to allow any relationship with surface hardness to be identified.

This study used a CIH to quantify surface hardness. For each location, the average of the second to fifth impact was used to the estimate the hardness which, combined with a single experienced operator collecting the data across all test sessions,^17^ had the intention of maximising reliability in this measurement. There is a current lack of consensus on the total number of impacts to use at a location and which to use to estimate hardness. The method selected will affect the hardness results since earlier impacts have the effect of compacting the infill leading to a general trend of surface hardness increasing with successive drops.^18^ For the current pitch, this effect was quite small with an average increase in hardness between the first and second drops of 4.6% and between the second and fifth drops of 1.3%, presumably due to the relatively short fibres and limited infill depth. Hence, although the surface hardness data obtained here may not be directly comparable to other studies that used a 2.25-kg CIH with a different test methodology, the focus was to develop a methodology that would allow the data to be compared between test locations across the pitch and across test sessions within this study. Although the CIH has been widely used in AT testing,^5,19^ the load and loading rates do not necessarily represent that experienced during player–surface interactions. The properties of the predominantly polymeric materials that make up a 3G AT system are likely to lead to stress–strain behaviours that are dependent on the magnitude and rate of loading.^1^ Therefore, some care is required in extending the presented hardness data to infer potential effects on player performance and safety.

Surface hardness can influence player performance and perception of the surface and is thought to affect injury risk.^20^ A number of studies have quantified the biomechanical changes for running on surfaces of different, but consistent, stiffness;^12,13^ however, less is known about how a player responds to surfaces demonstrating substantial spatial variation in stiffness. Fleming et al.^10^ found that a difference in force reduction of 5% (as measured with the AAA) led to significant differences in players’ perception of a surface. Evidence relating surface condition to injury has predominantly been circumstantial^21^ and, therefore, it is difficult to predict how spatial variation in surface hardness would affect this. While it has been recognised as important to monitor surface properties, the effect that a change in the surface properties can have on the nature of the player–surface interaction remains poorly understood.^1^ However, the results of this study combined with the above evidence suggest that the magnitudes of spatial variation in surface hardness measured in this study are likely to be detectable by players, and further investigation related to their specific effects on their biomechanics and perception of the surface is warranted.

To conclude, the degree of uniformity of a playing surface is likely to influence play performance in terms of player–surface and ball–surface interactions as well as the player perception of the surface. A 2-fold order of magnitude spatial variation in surface hardness has been measured on a mid-aged 3G AT pitch, with the main contributing factors being the shockpad thickness and infill depth. This spatial variation was over twice the magnitude of the temporal variation over the full year cycle of testing. Further study is required to determine how typical these results are for other 3G AT systems and to assess what effect this level of spatial variation has on the player experience of the surface, notably play performance and safety. The knowledge gained from this study can be used to help maintain and optimise the play performance of a 3G AT pitch over its lifespan, for example, the role of maintenance in keeping the infill evenly distributed and in a de-compacted state in helping to minimise spatial variation in surface hardness.

## References

[1] Fleming PR. Artificial turf systems for sport surfaces: current knowledge and research needs. Proc IMechE, Part P: J Sports Engineering Technology 2011; 225: 43–64.

[2] Burillo P Gallardo L Felipe JL. Mechanical assessment of artificial turf football pitches: the consequences of no quality certification. Sci Res Essays 2012; 7: 2457–2465.

[3] FIFA. FIFA quality concept for football turf: handbook of test methods. Zurich: FIFA, 2012, pp.15–21.

[4] Severn KA Fleming PR Young C. The play performance of six water based field hockey pitches – spatial and temporal changes. In: Fleming PR Young C Dixon SJ. (eds) First international conference science, technology and research into sports surfaces. Loughborough: Loughborough University, pp.1–18.

[5] McNitt AS Landschoot PJ Petrunak DM. Evaluation of the playing surface hardness of an infilled synthetic turf system. Acta Hortic 2004; 661: 559–565.

[6] Alcántara E Gamez J Rosa D. Analysis of the influence of rubber infill morphology on the mechanical performance of artificial turf surfaces for soccer. Proc IMechE, Part P: J Sports Engineering and Technology 2009; 223: 1–9.

[7] Severn KA Fleming PR Dixon N. Science of synthetic turf surfaces: player-surface interactions. Sports Techn 2010; 3: 13–25.

[8] Meyers MC. Incidence, mechanisms, and severity of game-related college football injuries on FieldTurf versus natural grass: a 3-year prospective study. Am J Sport Med 2010; 38: 687–697. 10.1177/0363546509352464 20075177

[9] Clegg B. An impact testing device for in situ base course evaluation. In: Proceedings of 8th ARRB conference, Perth, WA, Australia, 23–27 August 1976, vol. 8, pp.1–6. ARRB Group Ltd, Vermont South, VIC, Australia

[10] Fleming PR Young C Roberts JR. Human perceptions of artificial surfaces for field hockey. Sports Eng 2005; 8: 121–136.

[11] Potthast W. Motion differences in goal kicking on natural and artificial soccer turf systems. Footwear Sci 2010; 2: 29–35.

[12] Ferris DP Louie M Farley CT. Running in the real world: adjusting leg stiffness for different surfaces. Proc R Soc Lond B: Bio 1998; 265: 989–994. 10.1098/rspb.1998.0388 PMC1689165 9675909

[13] Kerdok AE Biewener AA McMahon TA. Energetics and mechanics of human running on surfaces of different stiffnesses. J Appl Physiol 2002; 92: 469–478. 11796653 10.1152/japplphysiol.01164.2000

[14] Orchard J. Is there a relationship between ground and climatic conditions and injuries in football? Sport Med 2002; 32: 419–432. 10.2165/00007256-200232070-00002 12015804

[15] Takemura M Schneiders A Bell M. Association of ground hardness with injuries in rugby union. Brit J Sport Med 2007; 41: 582–587. 10.1136/bjsm.2007.035568 PMC2465405 17504786

[16] International Rugby Board. Regulation 22: standard relating to the use of artificial playing surfaces. Dublin: IRB, 2004.

[17] Twomey DM Otago L Ullah S. Reliability of equipment for measuring the ground hardness and traction. Proc IMechE, Part P: J Sports Engineering and Technology 2011; 225: 131–137.

[18] Twomey DM Ullah S Petrass L. One, two, three or four: does the number of Clegg hammer drops alter ground hardness readings on natural grass? Proc IMechE, Part P: J Sports Engineering and Technology. Epub ahead of print 17 September 2013. 228: 33–39.

[19] Carré MJ Haake SJ. An examination of the Clegg impact hammer test with regard to the playing performance of synthetic sports surfaces. Sport Eng 2004; 7: 121–129.

[20] Dura JV Hoyos JV Martinez A. The effect of shock absorbing sports surfaces in jumping. Sport Eng 1999; 2: 97–102.

[21] Dixon SJ Batt ME Collop AC. Artificial playing surfaces research: a review of medical, engineering and biomechanical aspects. Int J Sport Med 1999; 20: 209–218. 10.1055/s-2007-971119 10376475

[22] Hopkins W. A new view of statistics, www.sportsci.org/resource/stats/effectmag.html (1997, accessed 17 October 2013).

